# Local ATP Generation by Brain-Type Creatine Kinase (CK-B) Facilitates Cell Motility

**DOI:** 10.1371/journal.pone.0005030

**Published:** 2009-03-31

**Authors:** Jan W. P. Kuiper, Remco van Horssen, Frank Oerlemans, Wilma Peters, Michiel M. T. van Dommelen, Mariska M. te Lindert, Timo L. M. ten Hagen, Edwin Janssen, Jack A. M. Fransen, Bé Wieringa

**Affiliations:** 1 Department of Cell Biology, Nijmegen Centre for Molecular Life Sciences (NCMLS), Radboud University Nijmegen Medical Centre, Nijmegen, The Netherlands; 2 Department of Surgical Oncology, Erasmus MC, Rotterdam, The Netherlands; Health Canada, Canada

## Abstract

**Background:**

Creatine Kinases (CK) catalyze the reversible transfer of high-energy phosphate groups between ATP and phosphocreatine, thereby playing a storage and distribution role in cellular energetics. Brain-type CK (CK-B) deficiency is coupled to loss of function in neural cell circuits, altered bone-remodeling by osteoclasts and complement-mediated phagocytotic activity of macrophages, processes sharing dependency on actomyosin dynamics.

**Methodology/Principal Findings:**

Here, we provide evidence for direct coupling between CK-B and actomyosin activities in cortical microdomains of astrocytes and fibroblasts during spreading and migration. CK-B transiently accumulates in membrane ruffles and ablation of CK-B activity affects spreading and migration performance. Complementation experiments in CK-B-deficient fibroblasts, using new strategies to force protein relocalization from cytosol to cortical sites at membranes, confirmed the contribution of compartmentalized CK-B to cell morphogenetic dynamics.

**Conclusion/Significance:**

Our results provide evidence that local cytoskeletal dynamics during cell motility is coupled to *on-site* availability of ATP generated by CK-B.

## Introduction

Distinctly different processes like protrusion and retraction of narrow surface projections by astrocytes, development of invadopodia by malignant cancer cells, pathogen binding and formation of phagocytic cups by macrophages, and the generation of protrusive structures at leading edges of migrating or extending cells share that they are driven by extensive local remodeling of the actin cytoskeleton [1], [2]. Central to the cytoskeletal dynamics in these protrusive structures, commonly involving formation of lamellipodia or filopodia, is the highly regulated actin cytoskeleton assembly, controlled by a dynamic balance between the addition of ATP-bound G-actin to the plus (i.e. “barbed”) end of a filament, the dissociation of ADP-actin from the minus end of the polymer, and the retrograde flow of the actin filament bundle [3]–[5]. In this process there is tight coupling to the intrinsic ATP hydrolysis capacity of individual actin subunits in the F-actin filament. Ultimately, the dynamic sheet and finger-like shape alterations that are characteristic for lamellipodial and filopodial protrusions are determined by branching, cross-linking and bundling of the linear actin filaments. The tight spatial and temporal control of the formation and dissipation of these higher-order structures involves a broad set of actin-binding proteins, like filamins, Arp2/3, cofilin, profilin and capping proteins, all regulating specific steps of actin dynamics [4], [6]. In concert with this actin machinery, molecular motor proteins like the non-muscle myosin-II and -X generate the periodically contractile force and provide the cargo-transport role that is important for fate specification of lamellipodia and filopodia during cell migration and adhesion [7], [8].

For the dynamic polymerization and filament-bundling of the actin cytoskeleton and for the control of movement and force generation by associated non-muscle myosin ATPases appropriate spatiotemporal regulation of ATP supply is needed [5], [9], [10]. Indeed, the weight of evidence in various studies now argues in favor of a model in which up to 50% of cellular energy expenditure via ATP hydrolysis is required for actomyosin dynamics in cell types with a constantly changing morphology, like neurons, astrocytes and immune cells [11], [12].

The intensively fluctuating rate of ATP turnover in mammalian cells with a high energy demand requires a robust system for supply, storage and distribution of energy in the form of high-energy phosphate compounds [13], [14]. Besides by direct generation of ATP via glycolytic kinases, or OXPHOS activity in the mitochondrial network, cells also fulfill this need via adenylate kinase (AK)-catalyzed phosphotransfer between the γ and β phosphate groups of ATP and ADP, or by creatine kinase (CK)-catalyzed phosphotransfer between phosphocreatine and ATP [15]. CK and AK circuits demonstrate both redundancy and specialization in their ATP generating functions [16], [17].

As shown by experiments in muscle tissue of AK-knockout mouse models, AK activity has an important role in the control of energetic economy [18], and recently we found that local AK1 expression facilitates motility of fibroblasts [19]. Muscle-type CKs (CK-M) have an important role in the control of contractile burst activity via maintenance of optimal local ATP/ADP ratios close to myosin ATPases in the contractile apparatus or by providing Ca^2+^-ATPases preferential access to ATP for calcium sequestration [20], [21]. Another CK, the brain-type isoform (CK-B, expressed in a range of cell types), may have similar roles in fueling of ATP-dependent cytoskeletal processes in non-muscle cells like oligodendrocytes, astrocytes, macrophages, osteoclasts and tumor cells, Functionally, CK-B is connected to spatial memory acquisition and behavior, development of the hippocampus, functioning of hair bundle cells in the auditory system, phagocytosis and bone resorption [22]–[27]. Despite extensive studies, the exact mechanisms by which CK-B activity controls these processes are still incompletely understood. A role in direct coupling of ATP supply to actin-myosin dynamics is by far the most likely mechanism since cytoskeletal activity is central to all processes named above.

Here, we provide evidence for a direct mechanistic connection between CK-B mediated phosphotransfer activity and local actin remodeling during cell migration and morphological changes. We show that the subcellular distribution of CK-B partially overlaps with that of dynamic actin in protrusion-active cortical areas of astrocytes and fibroblasts, but not with static F-actin of stress fibers in these cells. CK-B activity in astrocytes and fibroblasts directly facilitates actomyosin-driven motility, as demonstrated by cell spreading and migration assays. Additionally, by using deliberate positional swapping of CK-B from a cytosolic to membrane-bound location, we show that spatial confinement of CK-B activity controls local actin dynamics and therewith determines morphology and migration behavior.

## Results

### CK-B expression in cultured primary astrocytes

**Figure 1:**
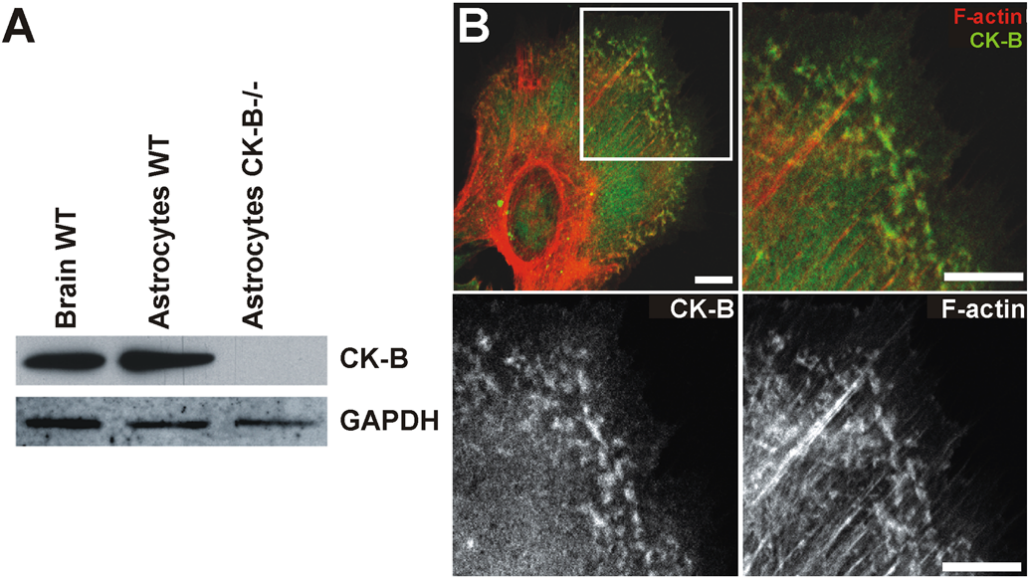
CK-B expression and localization in cultured astrocytes. (A) WT Brain tissue, cultured WT and CK-B knockout (CK-B−/−) astrocytes were analyzed for endogenous CK-B expression by Western blotting. GAPDH re-probing was used as protein loading control. (B) Astrocytes co-stained for CK-B (immunostaining) and F-actin (phalloidin). CK-B expression is cytosolic and additionally observed in peripheral patches that also stain positive for F-actin. Merge images of indicated panel revealed partial co-localization of CK-B and F-actin in peripheral patches but not in stress fibers. Lower panels show single stainings of same area. Bar, 10 µm.

Within the brain, CK-B is expressed at high levels in astrocytes [23], [28], which -in the *in vivo* context- are cells with highly dynamic surface extensions. We applied Western blot analysis to confirm that CK-B protein expression is also high in cultured primary astrocytes, *in vitro*. As anticipated, astrocytes derived from wild type (WT) mice gave a prominent signal, while control astrocytes derived from CK-B deficient mice [23] displayed no CK-B expression (Figure 1A). Immunostaining of CK-B in astrocytes revealed a diffuse cytoplasmic staining in most cells and strikingly, a number of cells displayed CK-B accumulation in peripheral patches. A magnification of the relevant cell area shows that these partially co-localize with phalloidin-stained actin. Interestingly, no overlap in localization was observed between the more prominent static actin stress-fibers and CK-B (Figure 1B).

### CK-B co-localizes with cortical actin and facilitates spreading of astrocytes

**Figure 2:**
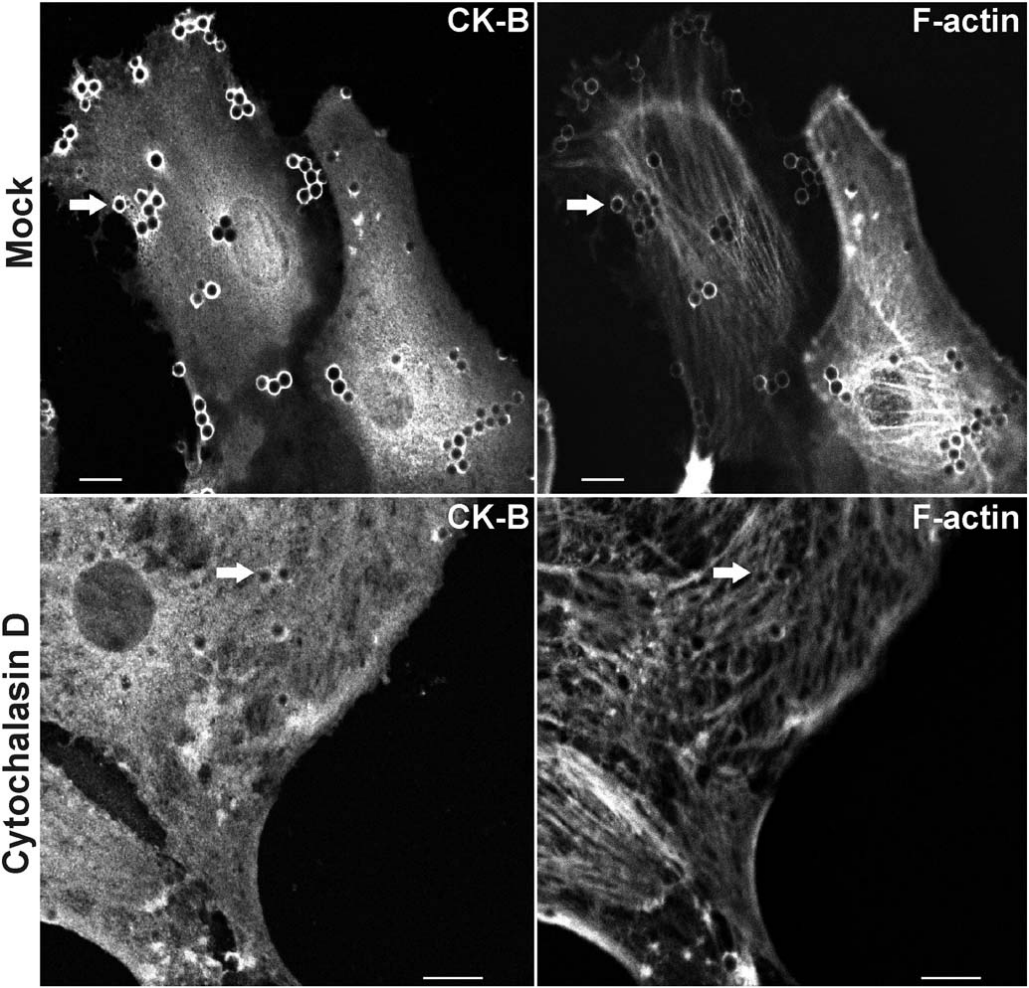
CK-B accumulation is F-actin dependent. WT astrocytes exposed to FN-coated latex beads were co-stained for CK-B (immunostaining) and F-actin (phalloidin). Both CK-B and F-actin accumulated around the beads (arrows). Cytochalasin D (10 µM) treated wild type astrocytes (lower panels) lack accumulation of CK-B and F-actin around FN-beads (arrows). Bar, 10 µm.

To study the possible coupling between CK-B accumulation and local actin dynamics in more detail, we analyzed astrocyte spreading in presence and absence of cytochalasin D, an inhibitor of actin polymerization. We took cell spreading as this is a process that is partially analogous to cell migration and dependent on actomyosin dynamics [29], [30]. During spreading, broad lamellipodial sheets and thin protrusions are formed, which are integrin- and receptor-rich and serve to probe the surrounding matrix and promote cell adhesion. Because cytochalasin D prevented astrocyte attachment in the conventional assay we used an approach by which fibronectin (FN)-coated beads were applied onto astrocytes as an “inverted adherence” assay [31]. In mock treated cells, CK-B and F-actin accumulated together at sites where FN-beads bound the cell membrane. In contrast, accumulation of F-actin and CK-B around beads was virtually absent in cells treated with cytochalasin D (Figure 2) confirming the coupling between local CK-B accumulation and local actin dynamics.

**Figure 3:**
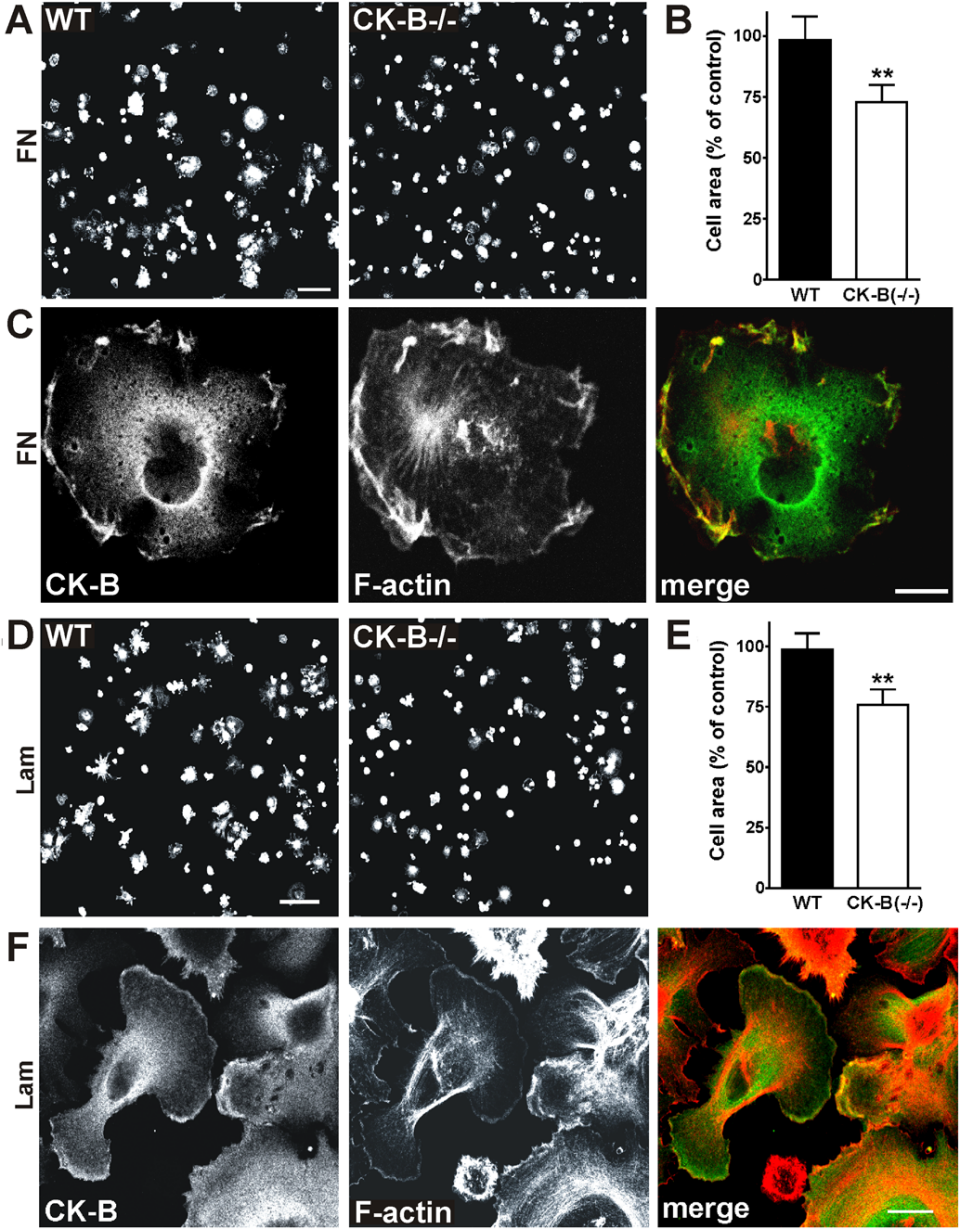
CK-B−/− astrocytes spread slower than WT astrocytes. (A) Astrocytes derived from WT and CK-B(−/−) mice were compared in quantitative cell spreading assays. Cells were seeded on FN and 30 min after seeding the cells were fixed and stained for F-actin. Bar, 100 µm. (B) Quantification of astrocyte spreading on FN represented as a percentage of the control (see materials and methods for details), ** p<0.01. (C) WT astrocytes spreading on FN co-stained for CK-B and F-actin. CK-B is distributed throughout the cytoplasm and accumulation is seen at membrane ruffles. Co-localization with cortical F-actin is shown in the merged image. Bar, 10 µm. (D) Quantitative cell spreading assay using WT and CK-B−/− astrocytes. Cells were seeded on Laminin (Lam) and 30 min after seeding the cells were fixed and stained for F-actin. Bar, 100 µm. (E) Quantification of astrocyte spreading on Lam showing decreased spreading capacities of CK-B−/− cells, ** p<0.01. (F) Astrocytes spreading on Lam co-stained for CK-B and F-actin. CK-B accumulation is seen at membrane ruffles. Co-localization with cortical F-actin is shown in the merged image. Bar, 10 µm.

To study the functional contribution of CK-B to astrocyte spreading, we performed cell spreading assays using WT and CK-B deficient (−/−) astrocytes. As shown in Figure 3, CK-B deficient cells consistently spreaded slower than their WT counterparts on both FN and laminin (Lam) although morphological appearances differed on both substrates. Under the experimental conditions used, the average area occupied by CK-B deficient cells was 27% smaller on FN and 24% smaller on Lam compared to WT cells (Figure 3B and E respectively; p<0.01; N = 3). Intriguingly, in most cells, a fraction of CK-B and F-actin jointly accumulated to ruffles at the periphery in actively spreading cells, i.e. in areas where actin-based structures are most dynamic (Figure 3C and F). It is of note, that for both matrixes we never observed co-localization of CK-B with actin stress fibers. We consider these data in support of a link between actin polymerisation dynamics and recruitment of CK-B activity in sub-cortical microdomains of astrocytes during spreading.

### CK-B enhances migration of astrocytes

**Figure 4:**
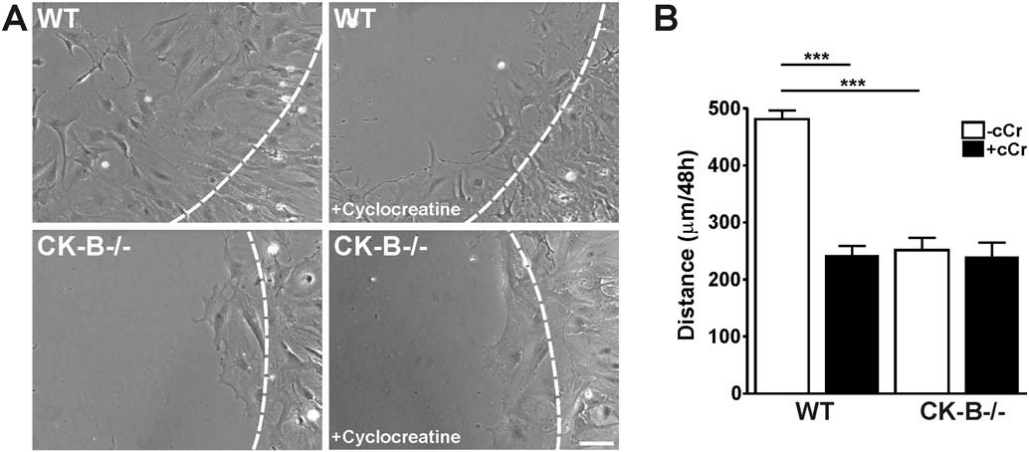
Astrocyte migration is dependent on presence and activity of CK-B. (A) Migration assays of WT and CK-B−/− astrocytes with and without cyclocreatine (5 mM) treatment. Representative images of migrating cells after 24 h, obtained from migration movies (Video S1). Dashed lines indicate migration front at 0 h. Bar, 100 µm. (B) Average migration distances of astrocytes migrating along laminin (10 µg/ml). Note that both CK-B presence and activity are required for migration, *** p<0.001.

Since spreading and migration events have analogous protrusive processes in common, we wondered whether astrocyte migration was also affected by absence or presence of CK-B. Using the barrier migration assay [32] we showed that WT-astrocytes migrated along FN matrices fairly efficiently (481±15 µm distance covered in 48 hr; Figure 4A and Video S1) but that astrocytes without CK-B migrated at 46% lower rate (252±22 µm distance covered in 48 hr, open bars in Figure 4B, p<0.001). Moreover, migration distances of WT-astrocytes decreased to similar levels as observed for knockout cells, when cells were treated with the specific CK-inhibitor cyclocreatine (closed bars in Figure 4B, p<0.001). We conclude that the difference in migration capacity is most likely directly attributable to the metabolic role of CK-B, i.e. its ATP generating capacity. Morphologically we did not observe major differences between migrating WT and CK-B deficient astrocytes.

### Subcellular targeting of CK-B affects migration and morphology in MEFs

Cell shape changes require highly coordinated activities of actin machinery that is localized (close) to the plasma membrane [4]. We designed a new experimental setting to test whether the observed recruitment of CK-B from the general cytosolic pool and concentration of its activity at cortical regions would serve ATP-dependent reactions directed to this machinery. Since primary astrocytes have a limited life-span and are inherently difficult to work with in permanent transfection protocols, we decided to use a complementation strategy in mouse embryonic fibroblasts (MEFs) from CK-B/AK1 double knockout mice (MEF-BAK−/−) to investigate the coupling between CK-B mediated phosphotransfer and regional cytoskeletal remodeling in greater detail.

**Figure 5:**
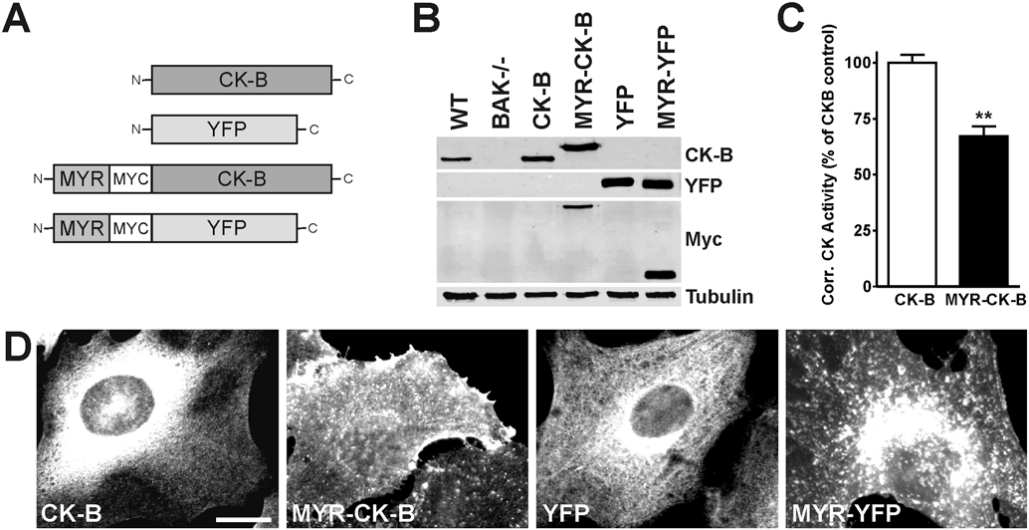
Generation of membrane-targeted CK-B expressing MEFs. (A) Constructs used to complement MEF-BAK−/− cells with CK-B and membrane-targeted CK-B (MYR-CK-B) and their corresponding YFP controls (not drawn to scale). (B) Western blot analysis of complemented MEF-BAK−/− cell lines. CK-B is seen in WT and (MYR)-CK-B complemented cells, YFP in YFP complemented cells, Myc in membrane-targeted CK-B and YFP, Tubulin was used as protein loading control. (C) CK-activity in whole cell lysates corrected for CK-B protein expression. Coupling of MYR-domain to CK-B renders the enzyme less active (approximately 70% of native CK-B), ** p<0.01. (D) Immunofluorescence showing the distribution of (targeted) CK-B and YFP in the generated cell lines. CK-B and YFP show a rather homogeneous cytoplasmic distribution while MYR-CK-B and MYR-YFP expressing cells showed staining of organellar and plasma membranes. Bar, 10 µm.

Activity of the ATP-generating enzyme AK1, which is expressed in MEF cells, is also linked to control of cell migration [19] and partly interchangeable with CK activity for offering fuel-demanding systems access to ATP [14]. Removal of both enzymes therefore gave us the opportunity to study effects of redistribution of enzymatic ATP/ADP exchange capacity without background effects. By re-expression of CK-B in MEF-BAK−/− cells were generated that express either cytosolic (native) CK-B or CK-B fused to a myristoylation (MYR)-tag, which provides a membrane-anchor function and (partly) redirects the enzyme to cellular membranes, thus enforcing accumulation of enzyme activity close to the sites with high cortical cytoskeletal plasticity. Control cell lines were generated by introduction of YFP variants (Figure 5A). Expression levels of native CK-B and MYR-CK-B were comparable between the two complemented cell lines and 2–3 times higher than that of endogenous CK-B in WT MEFs (Figure 5B). Importantly, CK activity in cells complemented with MYR-CK-B was about 30% lower than that in cells complemented with CK-B (normalized for CK-B expression levels; Figure 5C). This finding can be most easily explained by the observation that CKs expressed as N-terminal fusion proteins like β-galactosidase, GST or other peptide tags generally have a somewhat lower specific enzymatic activity (data not shown). Staining with CK-B specific antibodies or direct visualization of YFP fluorescence revealed a diffuse cytosolic distribution for CK-B and YFP in the MEF-CK-B and MEF-YFP cell pools, as anticipated. Analysis of MYR-CK-B and MYR-YFP cell lines confirmed that a significant part of CK-B and YFP protein was located at plasma and organellar membranes (Figure 5D).

**Figure 6:**
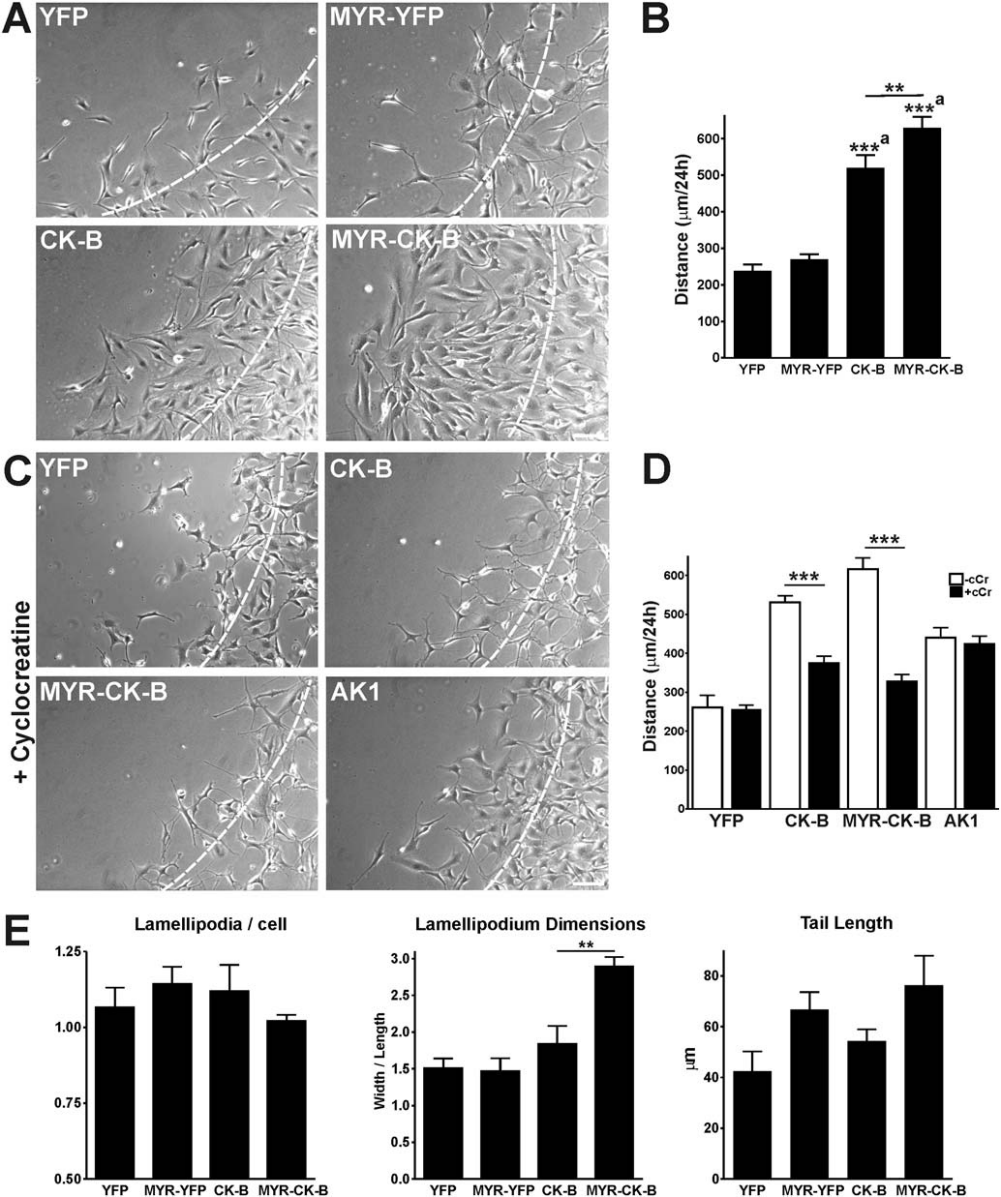
CK-B expression and localization increases fibroblast migration. (A) Morphology of migrating MEFs. Pictures of migrating cells after 24 h obtained from migration movies (Video S2). Dashed lines indicate migration front at 0 h. Bar, 100 µm. (B) Quantification of migration distances. MEF-CK-B and MEF-MYR-CK-B cells have higher migration capacities compared to the YFP-controls. Membrane targeting of CK-B further induces cell migration. Average migration distances in 24 h are shown, (a) CK-B and MYR-CK-B compared to matched YFP controls, *** p<0.001, ** p<0.01. (C) Morphology of migrating MEFs with cyclocreatine (cCr, 5 mM) treatment. Pictures of migrating cells after 24 h obtained from migration movies (Video S3). Dashed lines indicate migration front at 0 h. Bar, 100 µm. (D) Migration distances after 24 h of MEFs migrating along FN with (closed bars) and without (open bars) cyclocreatine (cCr, 5 µM). MEF-CK-B and MEF-MYR-CK-B cells respond to cCr treatment while MEF-YFP and MEF-AK1 (van Horssen et al., 2009) control cells do not. *** p<0.001 compared to non-treated cells. (E) Quantification of migration morphology. The number of lamellipodia per cell, lamellipodium dimensions and tail length were measured from migration movies. No differences were found for the YFP and MYR-YFP complemented cells. MYR-CK-B complemented cells showed an increased lamellipodium width/length ratio compared to CK-B complemented cells (** p<0.01). Measurements explanation and high magnification images of migration fronts are depicted in Figure S2.

Migration analysis (presented in Figure 6A (left panels) and Figure 6B) showed that re-expression of CK-B doubled the fibroblast migration capacity as compared to YFP-complemented controls (513±23 vs. 246±33 µm; p<0.001). This result corroborates the astrocyte migration data and underscores that our findings on CK-B effects can be extrapolated to other cell types. Interestingly, anchoring of CK-B to membranes (MYR-CK-B), thereby mimicking the change in distribution that occurs naturally in motile cells with CK-B, had a clear stimulatory effect on motility (Figure 6A and B, Video S2). Migration distances in MYR-CK-B cells were increased by a factor 2.4 (623±28 vs 262±21 µm, p<0.001) when compared to control MYR-YFP cells and also significantly enhanced compared to cells with non-tagged CK-B (21%, p<0.01). As indicated above, this latter effect could be even underestimated, because overall enzymatic activity in cells with MYR-CK-B was 30% lower than in cells with native CK-B. Enzymatic activity of CK-B was indispensable since cyclocreatine completely blocked the gain in migration capacity in both CK-B expressing cell pools (Figure 6C and D, Video S3) without overt effects on viability or proliferation rate (Figure S1). It is of note here that the directionality of cell movements, being the ratio of the direct distance from start to end point to the total migrated distance, was similar for all tested cell lines (data not shown). Thus, migratory capacity is increased without changing the type of cell movement. Additionally, we observed that the morphological appearance of migrating MEF-MYR-CK-B differed from that of MEF-CK-B, and displayed a more flat morphology with broad lamellipodia. Quantification of the lamellipodium dimensions (width/length of lamellipodium including lamella, measured in µm after 24 h or migration, see Figure S2) revealed that lamellipodia in migrating MYR-CK-B cells were broader, but not longer (ratio = 2.9±0.2, p = 0.0017), compared to dimensions in cells with native CK-B, YFP or MYR-YFP (ratios of 1.8±0.3, 1.5±0.1, 1.5±0.2, respectively; See Figure 6E for quantification and Figure S2 for high magnification images of migration fronts). In contrast, the number of lamellipodia per cell and the tail length of migrating cells appeared unaffected by the presence or specific localization of CK-B. To determine if any of the observed functional consequences of altered ATP supply capacity are also associated with matrix adhesion and extension as seen in spreading astrocytes, we performed spreading assays with the MEF lines (Figure S3). We observed no change in spreading capacity in MEFs with native CK-B, but MEFs with MYR-anchored CK-B covered a 25% larger area than MEFs without CK-B or with native CK-B under the experimental conditions used, confirming the importance for local CK-B in actin-dependent cell processes.

### Inducible translocation of CK-B to membranes: motility and morphological analyses

**Figure 7:**
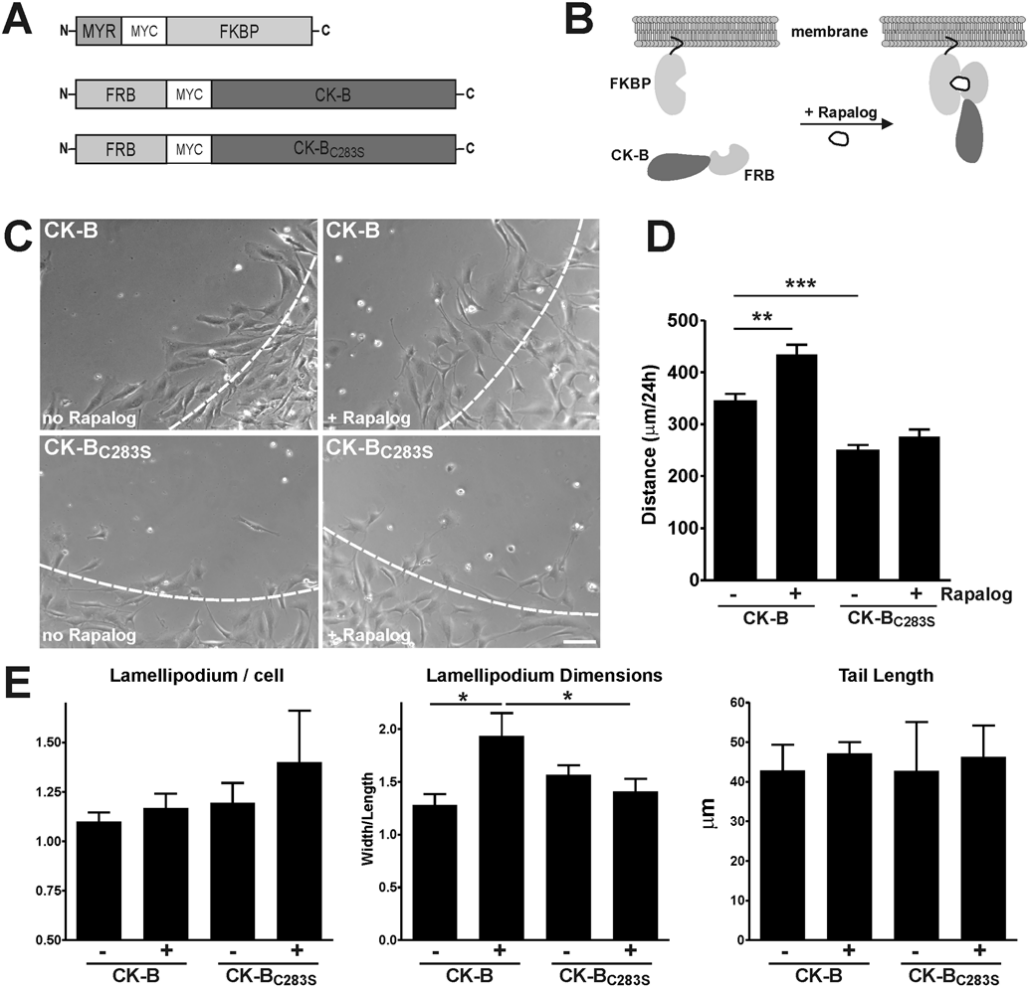
Rapalog-induced translocation of CK-B to membranes induced migration. (A) Constructs used to target CK-B, CK-B_C283S_ to cellular membranes. Vector inserts are not drawn to scale. (B) Scheme of heterodimerization of the MYR-FKBP fragment with FRB-CK-B on cellular membranes upon Rapalog addition. (C) Migration morphology of MEFs after 24 hr in absence or presence of Rapalog (Video S4). Dashed lines indicate migration front at 0 h. Bar, 100 µm. (D) Quantification of migration distances of MEF-MYR-FKBP complemented with FRB-CK-B or FRB-CK-B_C283S_ with and without Rapalog-treatment. Rapalog-induced translocation of FRB-CK-B, in contrast to FRB-CK-B_C283S_, enhanced cell migration. *** p<0.001, ** p<0.01. (E) Quantification of migration morphology. The number of lamellipodia per cell, lamellipodium dimensions and tail length were measured from migration movies. Lamellipodium dimensions for FRB-CK-B with Rapalog-treatment was significantly changed. * p<0.05. See Figure S4B for high magnification images of migration fronts.

Finally, we sought to uncouple effects of enzyme location from effects of enzyme level and activity in an independent approach. We reasoned that analyzing effects of manipulation of the intracellular spatial distribution of CK-B within the same cell would provide the most effective strategy. Therefore, we used the rapamycin dimerizer system [33] and engineered pools of MEF-BAK−/− cells with the constructs for inducible targeting of CK-B that are shown in Figure 7. One vector was designed for expression of FK506-binding protein (FKBP) with a MYR-anchor for targeting to membranes (MYR-FKBP). Other vectors were destined for expression of active CK-B or CK-B_C283S_, a catalytic-dead protein, both with N-terminal extensions consisting of the FK506-Rapamycin Binding (FRB) domain. Upon addition of Rapalog (rapamycin analog AP21967), inducible translocation of a significant fraction of cytosolic distributed CK-B and CK-B_C283S_ to membranes occurred, as verified by immunofluorescent staining (Figure S4A). Results of analysis of migration capacity of different MEFs in the presence and absence of Rapalog are shown in Figure 7C and Video S4. Quantification of video data showed that cells expressing native CK-B migrated significantly faster than cells expressing the enzymatically inactive CK-B_C283S_, independent of Rapalog-mediated induction. Secondly, when Rapalog was added during migration of cells with enzymatically active CK-B to redirect (part of) CK-B to membranes, distances increased from 344±15 to 434±19 µm over the period measured (3 independent experiments, p<0.01). Addition of Rapalog did not alter locomotion of CK-B_C283S_ expressing cells (Figure 7D). Together, these results indicate that covalent or interactive fusion of CK-B with FRB or FRB-FKBP does not impede CK-B intrinsic capacity to promote cell migration, and corroborate our finding that cell cortical presence of functional activity and not the mere structural presence of CK-B protein is essential (Figure 6). Since we noted earlier that permanent targeting of CK-B to membranes changed the lamellipodium dimensions in MEFs, we also assessed whether morphological changes would be inducible with Rapalog-treatment (Figure 7E and Figure S4B). Rapalog addition to CK-B MEFs caused a significant increase in the ratio of lamellipodium dimensions to 1.9±0.2, compared to 1.3±0.1 in non-treated cells (p = 0.0219). Importantly, Rapalog did not show this effect in MEFs expressing CK-B_C283S_, demonstrating that this effect solely depended on relocalisation of the enzymatic activity of CK-B and was not caused by non-specific effects of CK-B binding or the Rapalog treatment itself.

## Discussion

Actin-driven processes need considerable amounts of ATP to facilitate cytoskeletal dynamics. ATP exchange and hydrolysis is thereby critically involved in the process of actin treadmilling, controlled by recycling of G-actin and the stability of F-actin [3], and is also important for the activity of many actin-associated proteins. In the present study, we investigated the contribution of local ATP generation by CK-B to actomyosin dynamics by measuring spreading and motility of astrocytes and MEFs. Straightforward immunofluoresence and Western blot assays demonstrated that cultured astrocytes from mouse brain express high levels of CK-B, which corroborates findings with immunostaining and in-situ hybridization on whole mouse brain that appeared in other reports [23], [34]. Furthermore, it was demonstrated that astrocytes can synthesize creatine endogenously suggesting that a functional CK phosphoryl-transfer system is present in these cells [35], [36]. Recently, an emerging role of astrocytes as active modulators of synaptic transmission has been acknowledged. In this process, which depends on the intimate physical interactions between astrocytes and neurons, local actin-driven dynamics of astrocytic protrusions play an organizing and maintaining role [37], [38]. Based on this knowledge, we propose that astrocytes use a significant amount of their ATP for cytoskeletal remodeling, comparable to the ATP need for actomyosin activity in neurons [11].

Besides astrocytes, we also used mouse embryonic fibroblasts (MEFs) as archetypal motile cells for study of the localized role of CK-B in cell dynamics. Our data on recruitment of CK-B from the cytosolic pool and it's transient co-accumulation in areas with cortical F-actin but not with the less dynamic F-actin in stress fibers in both cell types support the idea that CK-B activity is generally most needed at sites where the actin cytoskeleton is highly dynamic. This idea was already proposed for the accumulation of CK-B in nascent phagosomes as well [24]. Interesting parallels can also be drawn to the fate of the muscle specific isoform of CK, CK-M, which is strategically localized in the M-band of myofibrils to support localized myosin ATPase activity [39], or is recruited to the I-band by binding to the glycolytic enzymes phosphofructokinase and aldolase [40]. CK-B lacks essential lysine residues that are required for CK-M accumulation at the M-band [41], but in view of its high homology with CK-M, it is tempting to speculate that other mechanisms for recruitment may be shared between the two enzymes. Especially, the possible interaction with glycolytic enzymes capable of binding to F-actin should be studied further as several of these enzymes are enriched in lamellipodia [42] and other types of dynamic cell structures [43]. We know already that CK-B and CK-M have an interchangeable character with respect to recruitment in the phagocytic cup of macrophages (data not shown), but the precise mechanistic link between protrusion formation and CK-B recruitment remains to be elucidated.

The direct functional link between CK-B activity and cell shape changes was clarified by our cell spreading and migration assays, in which ablation of CK-B slowed down these processes in both astrocytes and MEFs. Overall, findings between astrocytes and MEFs were comparable, only CK-B effects on spreading were not reproduced in MEFs. Cell spreading is a complex process consisting of substrate adherence and passive-descent events, later followed by active probing of the cellular environment [44]. These later events in spreading may be more reliant on infrastructural organization within the cell and be cell-type dependent. The observed discrepancies between the roles of soluble and anchored CK-B in spreading of astrocytes and MEFs may therefore be explained by differences in the relative importance of CK-mediated ATP supply during the early and late phases, and be not easily comparable between cell types.

Importantly, the experiments with pharmacological inhibition with cyclocreatine or substitution of native CK-B by the catalytically dead CK-B_C283S_ in the positional swapping/tagging experiments demonstrated that the contribution of CK-B to enhanced cell migration capacity did not depend merely on its structural interference, but that its catalytic ATP-generating activity is required.

We propose that cells use CK-B to balance ATP/ADP ratios and buffer temporary demands for ATP in specialized structures with highly dynamic actin, such as lamellipodia and filopodia, to sustain optimal migration rates. The early observation that cyclocreatine was able to inhibit motility in tumor cells [25] and the long known importance of ATP for actin polymerization and myosin force generation [5], [7] support this model.

One most conspicuous finding in our studies was that manipulation of CK-B location had an effect on both migratory capacity and lamellipodial morphology in MEFs. Interestingly, targeting CK-B to membranes changed the lamellipodial dimensions. Formation of lamellipodia is largely dependent on branched actin polymerization at the cell's periphery, which requires the availability of free ATP-loaded actin monomers [3], [5]. The process of re-loading of G-actin with ATP and incorporation of actin monomers at linear-barbed or branched-ends of polymeric filaments could be the decisive step that takes advantage of local ATP generated by CK-B. Our experimental verification of this obviously speculative model showed that CK-B with a membrane anchor could indeed facilitate lamellipodia formation and stimulated cell migration. Interestingly, expression of native cytosolic CK-B had an effect on migratory capacity but did not enhance lamellipodium formation proper. This suggests that other ATP dependent migration promoting processes, which are morphologically more difficult to delineate (and therefore association with local CK-B recruitment goes undetected) may also be fuelled by CK-B. Although speculative, actin mediated adhesion [45] or myosin force generation [7], [46] may be examples of such directly served processes. Earlier it has been suggested that CK-B may provide bursts of site-specific high-energy phosphate for signal transduction involved in cytoskeletal reorganisation [26]. In this latter process association of CK-B to the C-tail of protease activated receptor-1 (PAR-1) is involved. Whether similar stimulating activities of CK-B at other cellular locations would also require regional accumulation of the enzyme is completely unknown.

By using the Rapalog heterodimerizer system, we were able to manipulate the localization of CK-B without altering the total cellular levels of CK-B, thus excluding effects of variation in activity levels or in genetic or metabolic background between cells in our pool. As rapamycin is reported to inhibit F-actin reorganization [47], we verified that Rapalog (a chemically modified derivative of rapamycin) itself did not influence cell motility (data not shown). Our findings thus clearly indicate that migratory capacity and cell morphology are truly directly responsive to the localization of CK-B underscoring the importance that site-specific availability of ATP has for local cytoskeletal dynamics.

Besides CK-B, also other enzymes have been reported to act as local distributors of ATP at actin-rich structures. For example, translocation of adenylate kinase (AK1) to focal contacts sites in MEFs induced migratory capacity of these cells [19]. Other regulators of nucleoside-triphosphates levels (NTPs), such as nm23-H2 and arginine kinase, translocate to actin-rich cellular protrusions and lamellipodia and regulate their dynamics [48], [49]. As yet other examples, glycolytic enzymes form complexes that partition at specific subcellular locations such as pseudopodia [42], actin filaments [50] and phagosomes [51] and may have an energy supply role there. In Drosophila flight muscle, glycolytic multienzyme complexes provide myosin ATPases with preferential access to ATP when brought in close vicinity by physical protein-protein interactions [52]. Different cells may therefore use different systems to fulfill the ATP needs of active actomyosin.

In summary, data presented here strongly suggest that locally generated ATP is an important regulator for actin-based cytoskeletal dynamics involved in cell extension and motility and that CK-B is a controlling enzyme in the compartmentalization of ATP availability. This model also provides a plausible explanation for our earlier findings on synaptic function and development of neural cell networks in CK-B knockout mice [23], on epithelial hair cell function in the inner ear of [27], and on impairment of bone remodeling activity of osteoclasts [22] of these mice. In an analogous study we recently showed that CK-B fuels local actin dynamics in phagocytosis [24]. All processes mentioned have in common that they are dependent on the functional integrity and timely control of actin filament nucleation, elongation and associated motor protein activity in cell cortical regions that forms ruffles or membrane tubulation areas. More research is required to investigate whether CK-B also contributes to inhomogeneity in ATP distribution and whether the precise mechanistic events that connect CK-B mediated ATP supply with actin in this axis are shared between different CK-expressing cell types.

## Materials and Methods

### Cell culture and generation of cell lines

Primary cultures of astrocytes were established from brains of embryonic wild type (C57BL/6×129Ola) or CK-B deficient mice [23] at embryonic day 17 (E17) as described [53]. Astrocyte identity was verified by immunofluorescence staining for the astrocyte marker glial fibrillary acid protein (GFAP) and cells were cultured for 2-3 weeks in Dulbecco's Modified Eagle Medium (DMEM, Gibco) supplemented with 4 mM glutamine, 2 mM sodium pyruvate, 10% Fetal Calf Serum (FCS) and 0.5 mg/ml gentamycin. Mouse embryonic fibroblasts (MEFs) and Phoenix (HEK293) packaging cells were maintained in the same medium without gentamycin. MEFs were derived from 14.5 days old wild type (C57BL/6×129Ola) or CK-B/AK1 (BAK−/−) double knock-out mouse embryos. Therefore, CK-B deficient mice [23] were first mated with Adenylate Kinase-1 (AK1) deficient mice [18] and offspring was crossed again. F2 embryos were genotyped and lack of CK-B and AK1 was confirmed by Western blot and zymogram analysis. MEFs derived from the double knock out (MEF-BAK−/−) embryos were immortalized using established techniques [54] and used for retroviral complementation studies.

### Retroviral constructs and transfections

cDNA encoding CK-B, myristoylation tagged CK-B (MYR-CK-B) and their YFP controls were cloned into the EcoR1/Xho1 sites of the retroviral transfection vector pLZRS-IRES-Zeo [55]. The MYR-localization tag was introduced 5′-upstream of the myc-tagged CK-B and YFP by PCR using primers containing BamH1 sites to amplify a 50 bp MYR-domain segment from AK1β cDNA [56]. Natural start codons in the CK-B and YFP ORF were substituted by alanine codons to ensure the use of the ATP start codon of the MYR-domain. Inducible translocation of CK-B to membranes was studied using the ARGENT Regulated Heterodimerization Kit (ARIAD Pharmaceuticals), based on the rapamycin-induced heterodimerization of FKBP- and FRB-tagged proteins. The FKBP fragment (316 bp) from the pC4EN-F1 plasmid (ARIAD) was PCR-amplified, provided with EcoRI/XhoI sites, and cloned into the pLZRS-IRES-Zeo behind the MYR-myc sequence to generate the targeting construct. Subsequently, a 276 bp FRB fragment was PCR-amplified from plasmid pC4-RhE (ARIAD) with primers containing BamHI/BglII sites and cloned into the BamH1 site upstream of the myc-tagged CK-B or CK-B_C283S_ (catalytic death mutant) sequence in vector pLZRS-IRES-Zeo. Constructs and targeting principle are depicted in Figure 6 A and 6B, respectively. To generate retrovirus, Phoenix cells grown on poly-L-lysine (10 µg/ml) coated 6-wells plates were transfected with retroviral pLZRS constructs (5 µg) using Lipofectamine 2000 (10 µl, Invitrogen) diluted in Optimem (Gibco) according to the manufacturers protocol. Viral medium was harvested, polybrene (5 µg/ml) was added, filtered and used to infect MEF-BAK−/− cells grown to 40% confluence. Mixed pools of MEF-BAK−/− cells that co-expressed proteins with FKBP or FRB moieties were generated by sequential retroviral transduction. After 24 h, medium was replaced by medium containing zeocin (450 µg/ml) and cells were cultured under selection for at least two weeks. Cells were analyzed for protein expression by western blotting and immunofluorescence.

### Western blotting

Astrocyte cell lysates were prepared in lysisbuffer (12.5 mM Na_2_HPO_4_, 2.8 mM KH_2_PO_4_, 0.05% Triton X-100, 0.3 mM DTT) containing protease inhibitor cocktail (Roche). After incubation on ice (20 min) the cell lysates were centrifuged for 10 min at 13000 rpm and supernatants were collected. MEFs were lysed directly in sample buffer or in NP-40 Buffer (50 mM Tris-HCl, pH 7.5, 100 mM NaCl, 5 mM MgCl_2_, 1% NP-40, 1 mM PMSF and 1× protease inhibitor cocktail) and protein content was determined using the Bradford method. Samples were resolved by 10% SDS-PAGE and blotted onto nitrocellulose membranes. Subsequently, the membranes were blocked with PBS containing 5% non-fat milk and blots were probed with anti-CK-B (21E10, 1∶2000) [34], polyclonal anti-CK-B, 1∶2000 [57] or anti-myc (1∶100, Developmental Studies Hybridoma Bank, University of Iowa) or anti-YFP, 1∶3000 [58], anti-GAPDH (1∶3000, Trevigen) or anti-Tubulin (1∶2000, Developmental Studies Hybridoma Bank, University of Iowa) for 1 hr at RT or overnight at 4°C. Antibodies were detected with HRP-conjugated goat-anti-mouse IgG (1∶10000, Jackson ImmunoResearch) and the signal was developed with the HRP substrate Lumi-Light system (Roche). Alternatively, blots were incubated with goat-anti-mouse IRDye800 (Rockland) and goat-anti-mouse Alexa680 (Molecular Probes) simultaneously and signals were detected using the Odyssey Imaging System (LI-COR Biosciences).

### Immunofluorescence

Cells were grown on glass cover slips, washed twice in PBS and fixed with 2% paraformaldehyde in PHEM buffer (25 mM HEPES, 10 mM EGTA, 60 mM PIPES, 2 mM MgCl_2_, pH 6.9). Subsequently, cells were permeabilized with 0.1% Triton X-100 or 0.1% saponin and incubated for 20 min in PBS containing 1% bovine serum albumin (BSA). Incubation with primary and secondary antibodies was done for 1 h and in between incubations the cells were washes thrice with PBS. Primary antibodies used were polyclonal anti-GFAP (1∶500, DAKO), monoclonal anti-CK-B (21E10, 1∶2000) and polyclonal anti-CK-B (1∶2000). Secondary goat-anti-mouse and goat-anti-rabbit conjugated to Alexa 488 or Alexa 568 (1∶500, Molecular Probes). F-actin was visualized by phalloidin conjugated to Alexa 660 or TexasRed (Molecular Probes). Samples were dehydrated in 70% and 100% ethanol, air dried and mounted onto microscope slides using mowiol. Samples were analyzed on a Biorad MRC1024 laser scanning confocal microscope using an oil immersion 60× objective or on an Axiovert 35M fluorescence microscope (Carl Zeiss) using 63× and 100× oil immersion objectives.

### Cell spreading assay

Glass cover slips were coated with laminin (40 µg/ml) or fibronectin (50 µg/ml) for 2 hours at 37°C. Cells were collected by centrifugation, washed in DMEM/1% BSA (fatty acid free) and incubated in DMEM/1% BSA for 20 minutes at 37°C before seeding onto coated cover slips. After 30 minutes spreading on coated cover slips, cells were washed once with PBS and fixed in 2% paraformaldehyde. Cells were permeabilized and stained for CK-B and TexasRed conjugated phalloidin (Molecular Probes). For every cover slip 5–9 random fields were imaged with a Biorad Confocal Microscope MRC1024 using a 10× objective. The total surface occupied by cells was determined by ImageJ software (http://rsb.info.nih.gov/ij) and divided by the number of cells to calculate the area per cell. Five random fields were analyzed in each of three independent experiments. For every experiment the ratio between knockout and wild type was calculated (wildtype set at 100%).

### Cell migration assays and time-lapse microscopy

Barrier migration assays were performed as described previously [32]. Briefly, a coverslip was placed in an Attofluor incubation chamber (Molecular Probes) which was subsequently sterilized and coated with fibronectin. In this set up a removable, sterile circular migration barrier was placed creating a two compartment culture chamber which prevents cell growth into the middle of the coated cover slip. Cells were seeded around this barrier and grown until confluence. Subsequently, the migration barrier was removed and cells were washed twice before being incubated with the appropriate medium. MEFs were starved 4–24 h in DMEM containing 0,2% FCS and 5 mg/ml fatty-acid free BSA before bFGF (200 ng/ml, Peprotech) was added to stimulate migration. Astrocytes were analyzed in standard culture medium without starvation. Time-lapse imaging of migrating cells was done for 24 h in a Microscope Stage Incubator (Oko-Lab, Italy), ensuring optimal culture conditions, using a Nikon DiaPhot equipped with a Hamamatsu C8484-05G digital camera. Images were taken every 10 min using TimeLapse Software (Oko-Lab), version 2.7 with a 10× objective. Using time-lapse movies, cells were tracked taking nuclei as reference. For each treatment at least 8 migrating cells of 3–4 independent migration assays were analyzed. After cell division one of the daughter cells was followed. The total track distance (T) and the direct distance from start to end point (D) were measured. Measurements were done using Metamorph 6.2 software (Universal Imaging Corporation).

### Adhesion of fibronectin-coated beads to astrocytes

Polystyrene latex beads (3 µm, Sigma) were sonificated and coated by incubation in carbonate buffer (pH 9.6) with fibronectin (100 µg/ml) for 2 hours. The beads were washed twice in PBS and resuspended in serum free DMEM containing cytochalasin D (10 µm, Sigma) or DMSO as control before seeding onto cultured astrocytes. After incubation with FN-beads for 30 min at 37°C/5% CO_2_, cells were fixed and immunostained for CK-B and F-actin as described above.

### Proliferation and apoptosis assays

Proliferation rates and apoptosis of cyclocreatine-treated and control cells were determined using the WST-1 proliferation assay (Roche) and the Apo-ONE Homogeneous Caspase 3/7 Assay (Promega) according to manufacturers' protocols. MEFs were seeded at 5000 and 10000 cells per well for proliferation and apoptosis assays respectively. Proliferation was measured for time points op to 72 h. Apoptosis was measured in semi-confluent MEFs treated with cyclocreatine (5 mM, Sigma) for 24 h, staurosporin (0.5 µM, Sigma) was used as positive control.

### CK activity assay

CK activity was determined by enzyme coupled reactions using the Liquid NAC activated UV test (Human GmbH, Germany) according to manufactures' protocol. Cell lysates were prepared in NP-40 lysis buffer (50 mM Tris-HCL pH 7.5, 100 mM NaCl, 5 mM MgCl_2_, 1% NP-40, 1 mM PMSF, 1× protease inhibitor cocktail). Activity was normalized to CK-B content by performing quantitative western blotting in the same samples.

### Rapalog-induced membrane-targeting of CK-B

Translocation of CK-B from the cytosolic pool to cellular membranes within the same cell was done using the dimerization strategy based on the heterodimeration of FKBP and FRB protein domains by Rapalog [33]. MEF-BAK−/−, stably transfected with MYR-FKBP and non-transfected MEF-BAK−/− cells (control), were retrovirally transduced with FRB-CK-B or FRB-CK-B_C283S_ and directly (passage 2–8) used for further analysis. Rapalog-treated (200 nM, ARIAD) and untreated cells were analyzed for subcellular positioning of CK-B or CK-B_C283S_ and cell migration was monitored directly after Rapalog addition.

### Statistics

Data are presented as mean±SEM of at least three independent experiments. Groups were compared with Student's t-test for single values, one-sample t-test for relative values and with Ratio t-test for ratio-values and considered significant different when p<0.05.

## Supporting Information

Figure S1 Cyclocreatine does not alter proliferation and apoptosis. A) Proliferation of MEF-CK-B (left) and MEF-MYR-CK-B (right) cells with (red) and without (black) cCr (5 mM) treatment. Relative proliferation rates, taking non-treated cells as control, are shown of three independent experiments. (B) Apoptosis of MEF-CK-B and MEF-MYR-CK-B cells cultured without (open bars) and with (red bars) cCr (5 mM). Non-treated cells were set as 100% to compare cCr effects.
(3.90 MB TIF)

Figure S2 Morphology measurements of migrating MEFs. In the top panel, the analysis of migration morphology is illustrated. The number of lamellipodia per cell, the lamellipodium dimensions (width and length measured with cell body as reference, lamellipodium includes lamella-region) and tail length was measured. Lower panels show high magnification images of migration fronts of complemented MEFs and correspond to Figure 6A. Bar, 50 µm.
(2.72 MB TIF)

Figure S3 MEF-MYR-CK-B cells spread out faster than MEF-CK-B cells. Quantification of MEF spreading on FN for 30 min, showing that expression of MYR-CK-B facilitates cell spreading. * p<0.05.
(0.68 MB TIF)

Figure S4 Rapalog-induced membrane localization of CK-B and CK-BC238S in MEFs. (A) MEF-BAK−/− cells stably expressing MYR-FKBP were retrovirally transduced with FRB-CK-B (upper panels) or CK-BC283S (lower panels) and stained for CK-B. Rapalog treatment (100 nM, 1 h, left panels) resulted in translocation of (a fraction of) CK-B and CK-BC283S to cellular membranes. Bar, 10 µm (B) High magnification images of migration fronts are shown, corresponding to Figure 7C. CK-B (upper panels) and CK-BC238S (lower panels) without (left) and with (right) Rapalog treatment. Measurements were the same as shown in Figure S2. Bar, 50 µm.
(6.71 MB TIF)

Video S1 CK-B mediates astrocyte migration. WT (upper panels) and CK-B−/− (lower panels) astrocytes migrating along laminin for 48 h. Migration assays were performed without (left panels) and with (right panels) cyclocreatine treatment. Both CK-B knockout and inhibition strongly decreased migratory behavior of astrocytes. Video S1 corresponds with Figure 4A. Time-lapse phase-contrast microscopy was performed taking an image every 10 min. Display rate is 10 frames/s.
(9.22 MB MOV)

Video S2 CK-B expression and localization affects MEF migration. YFP/MYR-YFP complemented (upper panels) and CK-B/MYR-CK-B complemented MEFs migration along FN for 24 h. Migration assays of non-targeted (left panels) and membrane-targeted (right panels) cells showed that CK-B induced migration and additionally that MEF-MYR-CK-B were the most motile. Video S2 corresponds with Figure 6A. Time-lapse phase-contrast microscopy was performed taking an image every 10 min. Display rate is 10 frames/s.
(4.76 MB MOV)

Video S3 Cyclocreatine selectively inhibits CK-B-induced migration. MEF-YFP, MEF-CK-B, MEF-MYR-CK-B and MEK-AK1 cells were followed during migration along FN. In contrast to YFP and AK1 complemented MEFs, MEF-CK-B and MEF-MYR-CK-B migration was inhibited by cCr and a strong morphological effect was observed. Video S3 corresponds with Figure 6C. Time-lapse phase-contrast microscopy was performed by taking an image every 10 min. Display rate is 10 frames/s.
(7.47 MB MOV)

Video S4 Repositioning of CK-B to cellular membranes induces migration. MEF-MYR-FKBP cells transfected with FRB-CK-B (upper panels) or FRB-CK-BC283s (lower panels) were allowed to migrate along FN without (left panels) and with Rapalog (right panels). Rapalog-mediated targeting of CK-B to membranes, but not CK-BC283s membrane targeting, stimulated migration. Video S4 corresponds to Figure 7C. Time-lapse phase-contrast microscopy was performed taking an image every 10 min. Display rate is 10 frames/s.
(4.78 MB MOV)
