# Supplementary figures and images for: Local ATP Generation by Brain-Type Creatine Kinase (CK-B) Facilitates Cell Motility
Source: PLoS One. 2009 Mar 31;4(3):e5030. doi: 10.1371/journal.pone.0005030 (PMC2659440; doi:10.1371/journal.pone.0005030)

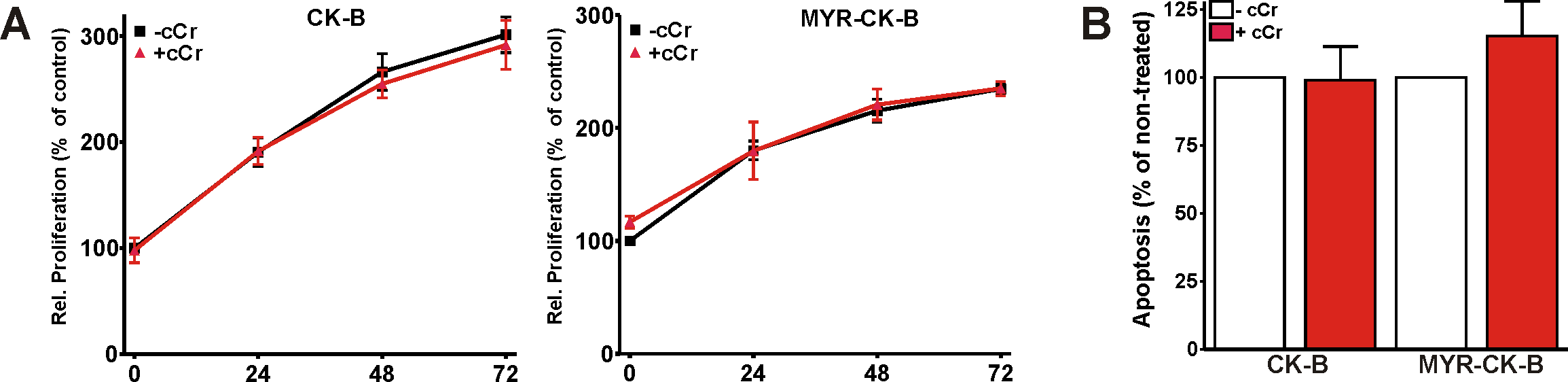

Supplement: Figure S1 — Cyclocreatine does not alter proliferation and apoptosis. A) Proliferation of MEF-CK-B (left) and MEF-MYR-CK-B (right) cells with (red) and without (black) cCr (5 mM) treatment. Relative proliferation rates, taking non-treated cells as control, are shown of three independent experiments. (B) Apoptosis of MEF-CK-B and MEF-MYR-CK-B cells cultured without (open bars) and with (red bars) cCr (5 mM). Non-treated cells were set as 100% to compare cCr effects. (3.90 MB TIF) [file pone.0005030.s001.tif]

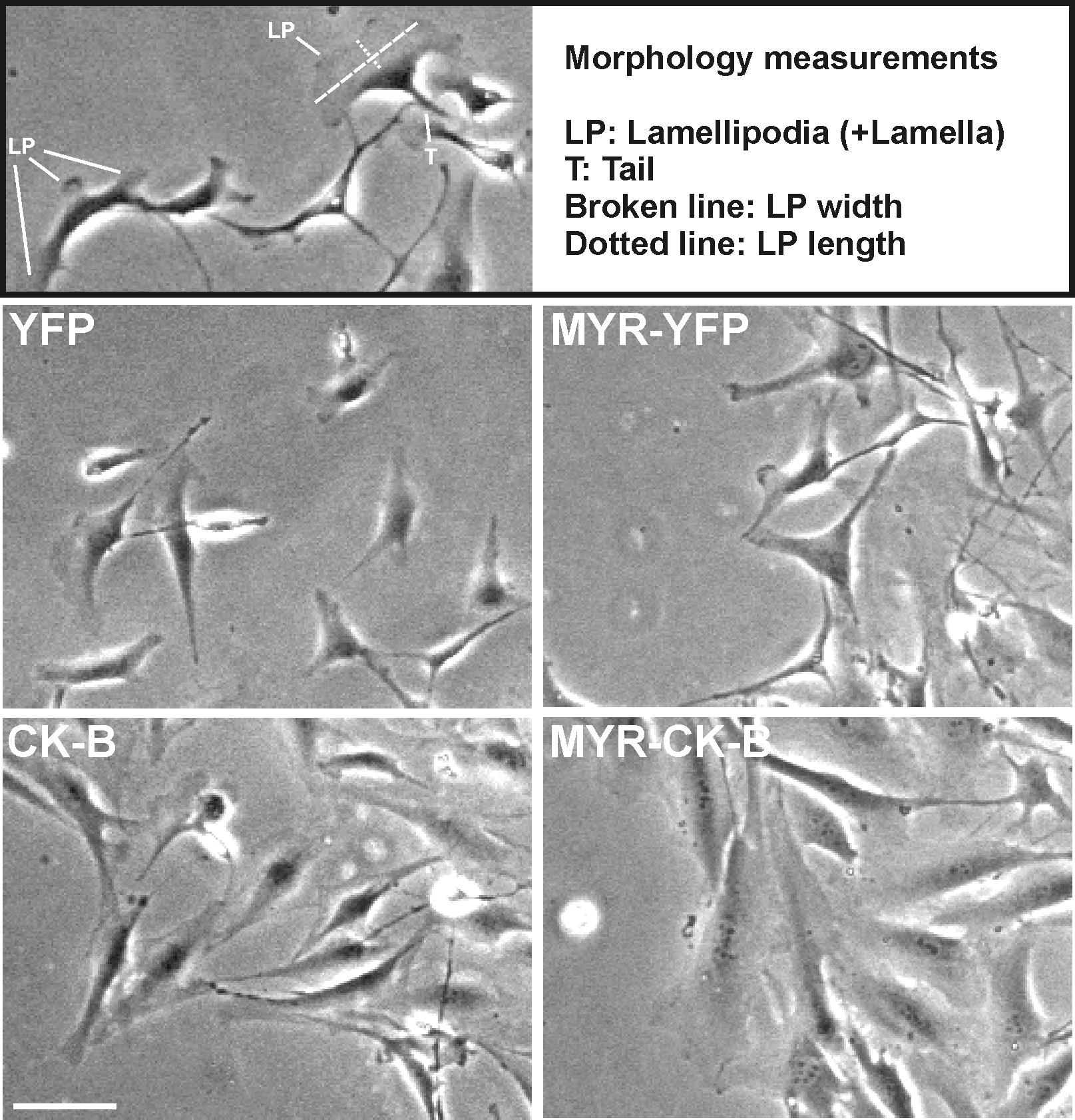

Supplement: Figure S2 — Morphology measurements of migrating MEFs. In the top panel, the analysis of migration morphology is illustrated. The number of lamellipodia per cell, the lamellipodium dimensions (width and length measured with cell body as reference, lamellipodium includes lamella-region) and tail length was measured. Lower panels show high magnification images of migration fronts of complemented MEFs and correspond to Figure 6A. Bar, 50 µm. (2.72 MB TIF) [file pone.0005030.s002.tif]

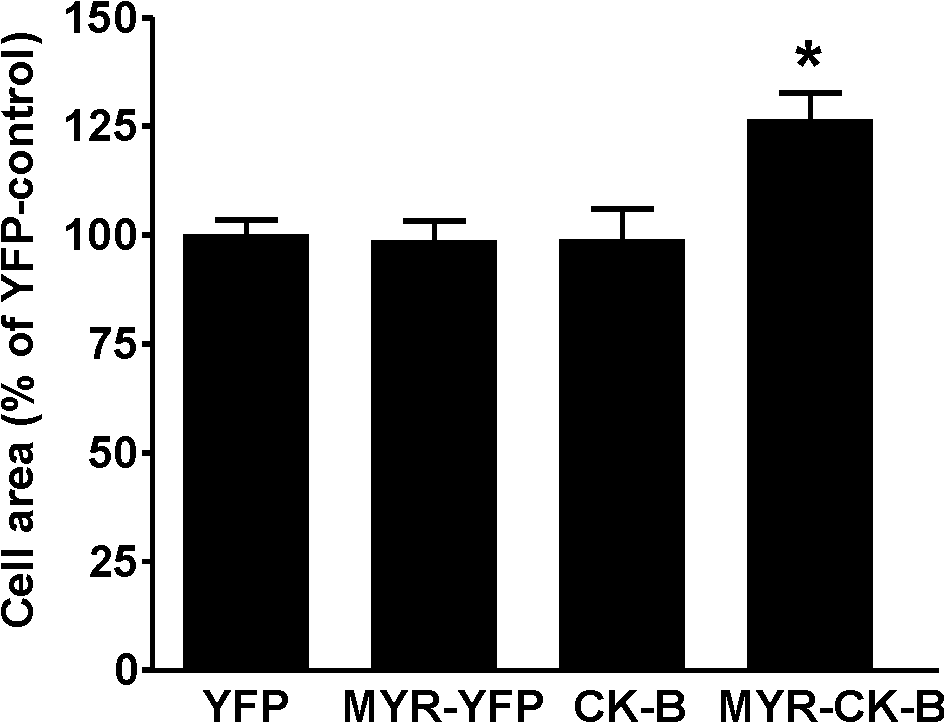

Supplement: Figure S3 — MEF-MYR-CK-B cells spread out faster than MEF-CK-B cells. Quantification of MEF spreading on FN for 30 min, showing that expression of MYR-CK-B facilitates cell spreading. * p<0.05. (0.68 MB TIF) [file pone.0005030.s003.tif]

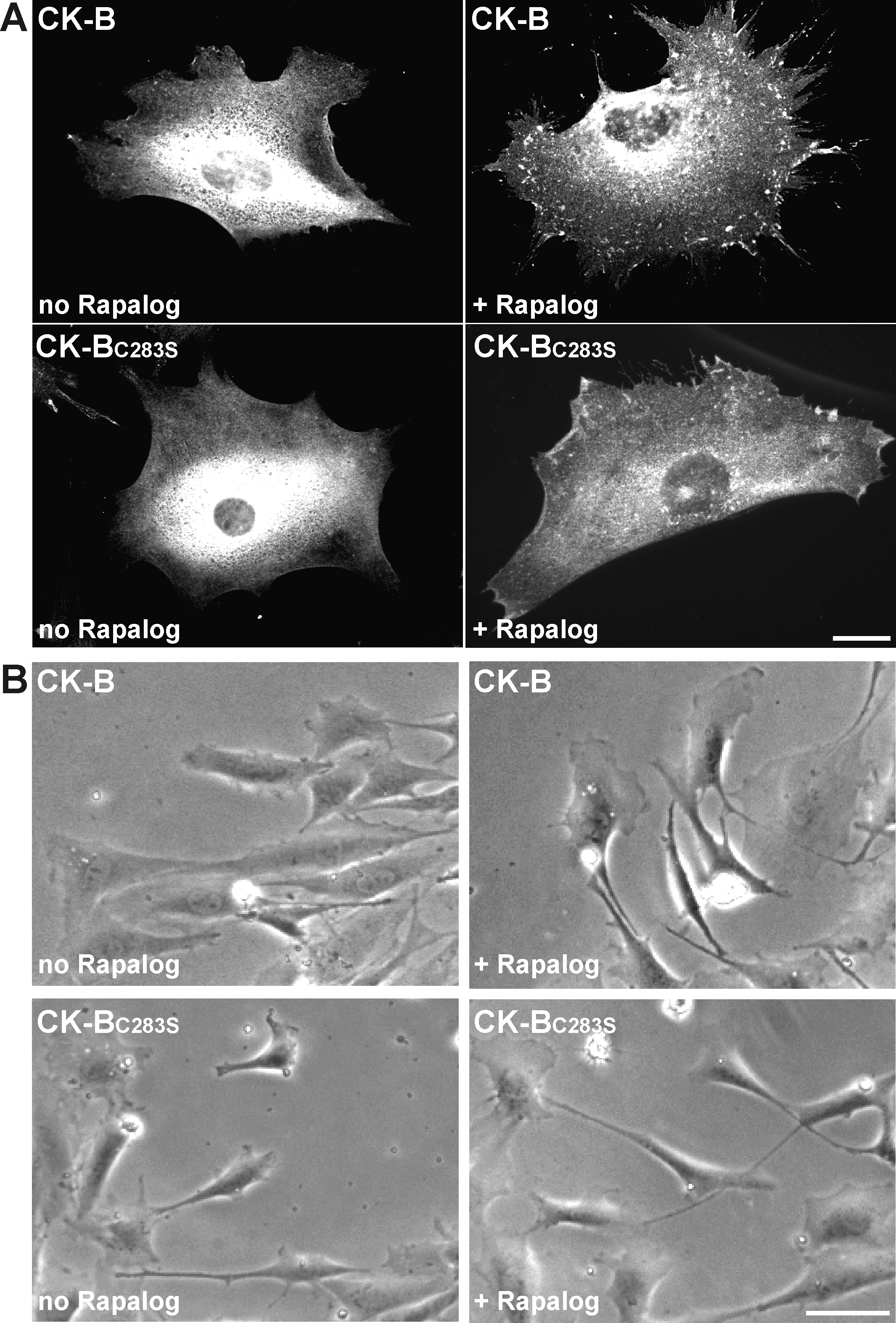

Supplement: Figure S4 — Rapalog-induced membrane localization of CK-B and CK-BC238S in MEFs. (A) MEF-BAK−/− cells stably expressing MYR-FKBP were retrovirally transduced with FRB-CK-B (upper panels) or CK-BC283S (lower panels) and stained for CK-B. Rapalog treatment (100 nM, 1 h, left panels) resulted in translocation of (a fraction of) CK-B and CK-BC283S to cellular membranes. Bar, 10 µm (B) High magnification images of migration fronts are shown, corresponding to Figure 7C. CK-B (upper panels) and CK-BC238S (lower panels) without (left) and with (right) Rapalog treatment. Measurements were the same as shown in Figure S2. Bar, 50 µm. (6.71 MB TIF) [file pone.0005030.s004.tif]
